# Gatekeeper or Pathfinder? The Evolving Role of Lymphedema Surgeons in the Assessment of Limb Swelling

**DOI:** 10.3390/jcm15041322

**Published:** 2026-02-07

**Authors:** Judith Monzy, Yasmina Samaha, Shelby Chun Fat, Eileen Lu, Christopher Pham, Edward C. Ray, Philip S. Brazio

**Affiliations:** 1 Division of Plastic and Reconstructive Surgery, Department of Surgery, Cedars-Sinai Medical Center, Los Angeles, CA 90048, USA; judith.monzy@cshs.org (J.M.); shelby.chunfat@cshs.org (S.C.F.); eileenlu99@gmail.com (E.L.); edward.ray@cshs.org (E.C.R.); 2 Division of Plastic and Reconstructive Surgery, Department of Surgery, Stony Brook University Hospital, Stony Brook, NY 11794, USA; yasminasamahamed@gmail.com; 3 Department of Orthopedic Surgery, University of California Los Angeles Health, Los Angeles, CA 90095, USA; christopherhapham@gmail.com

**Keywords:** lymphedema, lymphovenous bypass, vascularized lymph node transfer, lymphatic surgery, referrals, plastic surgery, microsurgery, supermicrosurgery, multidisciplinary care

## Abstract

**Background:** Lymphedema is a debilitating condition with high morbidity, yet despite advances in management, diagnostic ambiguity and fragmented referral patterns continue to delay appropriate care. We evaluated predictors of accurate diagnosis, microsurgical reconstruction candidacy, and secondary referrals generated during consultation with a lymphatic microsurgeon to highlight the need for a coordinated model of care. **Methods:** A retrospective chart review was performed for all outpatient referrals for ‘lymphedema’ from September 2020 to September 2021. Patient demographics, diagnostics, referral patterns, and lymphedema-related clinical data were collected. **Results:** 94 patients were referred for evaluation of possible lymphedema; lymphoscintigraphy confirmed diagnosis in 69. Following consultation, 23 patients received referrals for physical therapy, 17 for vascular surgery, and 8 for bariatric surgery or medical weight loss. Patients without lymphedema were more often referred to vascular surgery than those with lymphedema. Non-surgical candidates were more frequently referred to therapy or weight loss. **Conclusions:** Incorporating microsurgical reconstructive expertise into the evaluation of limb swelling improves diagnostic accuracy and refers patients- regardless of lymphedema status or surgical candidacy- to appropriate specialists. We propose a pathfinder model for patient flow that streamlines triage, improves access to accurate diagnosis and treatment, and prevents overburdening microsurgical practices with non-surgical patients.

## 1. Introduction

Lymphedema is the chronic, pathologic accumulation of interstitial fluid in tissues caused by dysfunction of the lymphatic system [1]. It is a progressive condition that initially presents with limb swelling and can eventually cause significant physical morbidity, decreased quality of life, and increased healthcare costs for affected individuals [2,3]. Lymphedema may be categorized, based on the cause of the lymphatic dysfunction, into primary and secondary types. Secondary lymphedema, with an estimated prevalence of 1:1000, results from injury to a previously intact lymphatic system [4]. In the United States, cancer-related lymphedema is the most common cause of secondary lymphedema, with reported incidences of approximately 25% following axillary lymph node dissection [5] and 11% following local radiation [6]. Globally, lymphatic filariasis and podoconiosis are the most common causes of secondary lymphedema [4]. Secondary lymphedema may also arise from non-cancer-related causes such as trauma, chronic venous disease, obesity, and genetic predisposition [4]. Primary lymphedema, which is less common with an estimated prevalence of 1:87,000 [7], arises from the aberrant development of the lymphatic system. It occurs most frequently in females and often presents sporadically, with lower morbidity than secondary lymphedema [4].

Early detection is crucial for patients with lymphedema, as there is no cure for this highly morbid condition and outcomes improve significantly with prompt intervention [1]. Advances in diagnostic tools have facilitated earlier identification of lymphedema. Nuclear lymphoscintigraphy, the most widely used diagnostic modality [1], uses a radiotracer to visualize areas of lymphatic dysfunction. Indocyanine green (ICG) lymphography offers real-time visualization of lymphatic flow and can assist with surgical planning [8]. Magnetic resonance lymphography provides excellent deep and superficial spatial resolution but is still not widely available [1]. Bioimpedance analysis (BIA) measures electrical impedance to estimate limb volume and assess fluid changes [9].

Surgical procedures have also evolved to address the physiology of the obstruction and optimize patient outcomes. The goals of surgery are to prevent disease progression, improve symptoms, decrease the self-care burden of compressive therapy and manual lymphatic drainage, and reduce the risk of infections [10]. Surgical techniques including lymphovenous anastomosis (LVA), vascularized lymph node transfers (VLNT), and liposuction have largely replaced open excisional procedures in the modern era of lymphatic surgery [11]. LVA and VLNT are physiologic procedures that restore lymphatic function by bypassing obstructed lymphatics and facilitating lymphangiogenesis, respectively [11]. Liposuction is a reductive procedure reserved for patients with fat-predominant lymphedema and requires lifelong adherence to compression therapy [11]. Physiologic procedures such as LVA and VLNT have been associated with reductions in excess limb circumference of up to 60% [12], decreased episodes of cellulitis, and improved quality of life in patients with secondary lymphedema [12,13,14,15,16]. Reductive procedures such as liposuction have demonstrated similar benefits, with more than 90% reduction in excess limb volume and subjective improvements in quality of life, especially when combined with LVA or VLNT [17].

Despite these advances, the general level of education and awareness regarding lymphedema diagnosis and treatment remains low among referring providers. Many continue to view lymphedema as a clinical diagnosis or a diagnosis of exclusion, unaware of available imaging modalities. Furthermore, the onset and progression of disease are highly variable, further complicating diagnosis [18]. Finally, lymphedema may coexist with other etiologies of limb swelling such as venous insufficiency or lipedema, contributing to diagnostic uncertainty [18].

The low level of provider education, coupled with the complexity of limb swelling disorders, creates a reproducible sequence of challenges across institutions [19]. First, lymphedema is frequently misclassified as a venous or adipose disorder, leading to both over- and under-diagnosis depending on clinician exposure to lymphatic disease. Second, limited awareness of confirmatory imaging results in either unnecessary testing or missed opportunities for early-stage identification. Third, the resulting influx of referrals for patients without true lymphatic dysfunction strains specialized surgical practices and delays consultation for appropriate candidates. The outcome is an inefficient system where surgeons serve as de facto diagnosticians, consuming time and resources that could otherwise be directed toward definitive treatment.

To avoid overwhelming a surgical practice with non-surgical candidates, some lymphedema centers employ referral criteria to pre-screen for patients with confirmed lymphedema or those who are likely to be surgical candidates, including prior cancer diagnosis, imaging-confirmed lymphedema, or body mass index cutoffs [20,21]. While this triage approach conserves resources for patients who are most likely to benefit, it may also exclude appropriate patients from evaluation by a lymphatic surgeon or specialist. Various vignettes may be imagined: tests are never ordered because of low referring provider education; a patient never completes testing because they become frustrated trying to find an imaging center that takes their insurance and can generate the appropriate diagnostic images; a patient with obesity-induced lymphedema (OIL) [22], whose lymphedema cure is weight loss, is never informed of the critical importance of referral for medical or surgical weight loss for their lymphedema. From this perspective, the low level of provider familiarity with diagnostic algorithms has consequences beyond workflow inefficiency. This perpetuates therapeutic nihilism among referring physicians and delays patient engagement with specialized care [23,24,25].

A rational model must balance referral triage and diagnostic accessibility to align patient flow with surgical capability. Plastic surgeons are uniquely positioned to address this gap by defining practical workflows and clarifying indications for physiologic surgery. We analyzed referrals to our practice for ‘lymphedema’ to characterize referral patterns, diagnostic accuracy, and subsequent management (‘secondary referrals’), with the goal of supporting an approach that allows lymphatic specialists to maintain access, treat non-surgical patients, and preserve the patient selection accuracy required for surgical success.

## 2. Materials and Methods

### 2.1. Study Design and Population

We retrospectively reviewed all referrals to our lymphatic surgery practice over its first year (September 2020 to September 2021) to characterize referral patterns, diagnostic accuracy, and subsequent management. Institutional review board approval was obtained with a waiver of informed consent for retrospective chart review. No pre-screening criteria were applied.

### 2.2. Diagnostic Evaluation

All patients referred for ‘lymphedema’ underwent an initial evaluation by the senior author (P.S.B.), performed in person or via telehealth when travel was not possible. The evaluation included a standardized history focused on lymphedema risk factors (i.e., history of radiation, prior infections, history of trauma), symptoms and their timeline, prior diagnostic testing performed, and prior conservative management (i.e., lymphedema-specific therapy, pump device usage, compression garment usage) trialed. This was followed by a physical examination that assessed limb asymmetry, distribution of swelling, edema characteristics, and skin changes. For telehealth visits, patients showed the senior author (P.S.B.) their affected extremity and performed a self-exam to demonstrate pitting vs. nonpitting swelling.

The patient’s nonoperative management regimen was reviewed. Patients not actively engaged in lymphedema therapy were referred to a certified lymphedema therapist. Diagnostic imaging was ordered, and always included lymphoscintigraphy and venous duplex if a recent study (<1 year) was not available. Lymphoscintigraphy served as the primary imaging modality for diagnosing lymphedema because it is reproducible, widely available, and can be obtained near the patient’s home. Venous duplex assessed for venous obstruction in the upper extremity, and venous obstruction or insufficiency in the lower extremity. Ancillary tests, such as interventional radiology lymphangiography, were ordered if patients demonstrated advanced disease, such as symptoms suggestive of disruption of the thoracic duct.

### 2.3. Diagnostic Criteria

The diagnosis of lymphedema was based clinical history, physical examination, and positive lymphoscintigram. The specific diagnostic criteria for each lymphedema category and non-lymphedema-related limb swelling category applied in our practice are summarized in Table 1.

Lymphedema was staged based on clinical presentation according to the International Society of Lymphology staging criteria. Imaging later confirmed the diagnosis of lymphedema. Patients with stage I lymphedema had slight pitting edema and swelling that subsided readily with elevation. Stage II lymphedema patients had more pronounced pitting edema that rarely subsided with elevation, due to the increased fibrofatty composition of the swelling. Stage III lymphedema patients displayed nonpitting edema unresolved with elevation, hyperpigmentation, hyperkeratosis, and verrucae.

The definitive diagnosis of lymphedema based on lymphoscintigraphy requires at least one positive finding: dermal backflow, decreased nodal uptake, slow progression of tracer to regional lymph nodes (>3 h), visualization of popliteal lymph nodes, and/or the presence of collateral lymphatic pathways [28].

Venous duplex US was classified as obstruction if noted by the interpreting radiologist or vascular surgeon. Lower extremity venous insufficiency was defined as 1000 ms or more of reflux in more than one segment of the deep venous system, or 500 ms or more of reflux in more than one segment of the superficial venous system.

### 2.4. Criteria for Microvascular Reconstruction Candidacy

Criteria for microvascular reconstruction candidacy were defined to identify patients most likely to benefit from surgery. Lymphedema patients were considered for microvascular reconstruction based on the following inclusion criteria:

(1) Lymphatic dysfunction resulting in persistent symptoms despite optimization of other conditions, such as venous insufficiency, lipedema, or obesity
(2) Patients with phlebolymphedema demonstrating lymphatic dysfunction and surgically correctable venous disease
(3) Obese patients with anatomic lymphatic obstruction on lymphoscintigraphy (not OIL)
(4) Patients with primary lymphedema

Some patients were identified as potential surgical candidates based on their clinical presentation and imaging findings but had not yet been optimized for surgery- undergone decongestive therapy, venous ablation, or weight optimization- and were therefore considered candidates contingent upon successful optimization of these non-microsurgical categories. Briefly, patients pursuing surgery completed a structured preoperative conservative treatment protocol starting six weeks before surgery, which consisted of compression garment wear and evaluation of self-care or caregiver needs. They then underwent around 4 weeks of complete decongestive therapy- consisting of manual lymphatic drainage, short stretch compression bandaging, exercises for lymphatic flow, appropriate skin care, and use of compression garments- to optimize limb volume and practice appropriate compression garment use. Compression garments are used postoperatively to maintain limb volume reduction.

Microsurgical reconstructive options offered for surgical candidates included lymphovenous anastomosis (LVA) and vascularized lymph node transfer (VLNT). Patients were deemed ineligible for microsurgical reconstruction based on the following exclusion criteria:

(1) Nonadherence or unwillingness to trial conservative management, including consistent compression garment use, pneumatic compression therapy, and physical therapy with a certified lymphedema therapist
(2) Lacking interest in surgical intervention
(3) Clinical or imaging findings suggestive of a non-lymphedema etiology, including venous occlusion, reflux, or insufficiency suggestive of chronic venous insufficiency, diffuse or localized swelling with an atypical history inconsistent with the diagnosis of lymphedema
(4) Substantial improvement with conservative therapy alone obviating the need for surgical intervention
(5) Active medical or oncologic contraindications, such as ongoing radiation therapy or limited life expectancy
(6) Surgically untreated lipolymphedema, for which liposuction was offered as the preferred intervention
(7) Phlebolymphedema with deep or non-correctable venous dysfunction, as demonstrated on duplex ultrasonography and/or venography

Patients with untreated OIL, surgically untreated lipolymphedema, or phlebolymphedema with deep or untreatable venous dysfunction (on duplex and/or venography) were deemed ineligible for microsurgical reconstruction. Patients with lipolymphedema were offered liposuction. Patients with OIL were defined by obesity, bilateral symptoms, and lymphoscintigraphy showing normal lymph nodes and primary channels with only peripheral or local dermal backflow. These patients were referred to bariatric surgery or medical weight loss as per their preference.

Data were analyzed using SPSS software version 24 (SPSS Inc., Armonk, NY, USA). Data collected included patient demographics, relevant medical history, referral source, chief complaint, previous therapies, diagnostic tests, clinical suspicion of lymphedema, lipedema, or venous insufficiency, secondary referrals (from the lymphedema specialist), and interventions planned by secondary referrals. Descriptive statistics reported for patient demographics and clinical characteristics (history of chronic venous insufficiency, sentinel lymph node biopsy, radiation therapy, lymph node dissection, upper and lower extremity symptoms, type of referral to the lymphedema surgeon, and secondary referrals) were compared using chi-squared tests. Continuous variables were compared using two-tailed t-tests. A *p*-value of <0.05 was considered statistically significant.

## 3. Results

### 3.1. Patient Characteristics

A total of 94 patients were referred for the evaluation of possible lymphedema. The majority of patients were female (72.3%). The mean age of patients was 61 years (range, 20 to 86 years). The average BMI was 29.8 kg/m^2^ (range, 21.8 to 53.8 kg/m^2^) (Table 2). 31 patients (33%) had a history of smoking- either current or former. Lower extremity complaints were most common (54.3%). 4 patients (4.3%) had upper and lower extremity symptoms (Table 2).

Of the patients who were referred for lymphedema, the majority experienced symptoms after direct injury to the lymph nodes from cancer treatment (54%). The majority of patients who received cancer treatment had cancer near the affected limb (54.3%) and underwent prior radiation therapy (44.7%) (Table 3). At the time of the initial consultation, 77.7% of patients had previously been seen by a certified lymphedema therapist (CLT), and over half (54.3%) were actively receiving conservative treatment (Table 3)**.**

### 3.2. Referral Source and Visit Type

Plastic and reconstructive surgery was the most common referral source (Table 4). 18.1% of patients were seen for initial consultation by video visit. There was no significant difference in any analyzed outcome between video-visit and in-person-visit patients.

### 3.3. Patients with Lymphedema vs. Without Lymphedema

**Figure 1:**
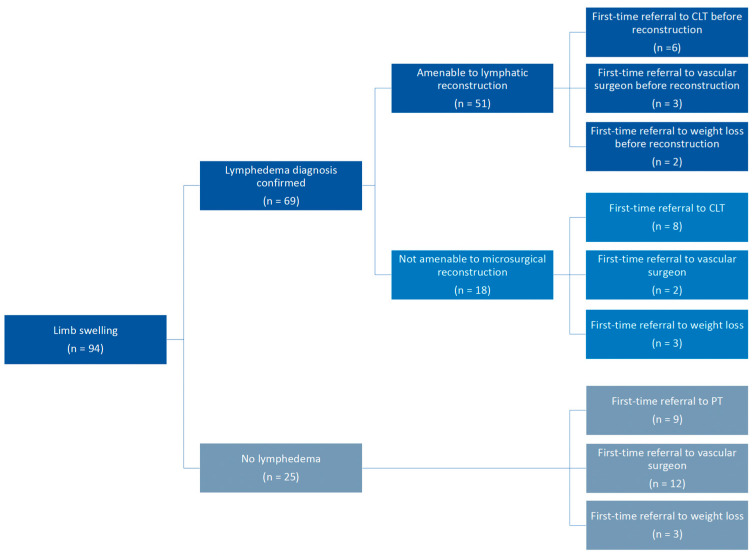
Characterization of patients referred for lymphedema, including diagnosis, eligibility for microsurgical lymphatic reconstruction, and referrals to other providers generated after consultation with lymphedema specialist. CLT: certified lymphedema therapist. PT: physical therapy.

Of the 94 patients referred, 69 (73%) had lymphedema, while 25 (26%) did not (Figure 1). Of the patients diagnosed with lymphedema, most had secondary lymphedema (81%), followed by idiopathic primary lymphedema (12.2%) and obesity-induced lymphedema (6.8%). Of note, none of the referred patients demonstrated syndromic features; therefore, genetic testing was not ordered. Those without lymphedema were diagnosed with lipedema, deep vein thrombosis, venous insufficiency, May-Thurner syndrome, obesity, lymphocele, tumor metastases, vasomotor disease, and axillary cording. There were no patients without an attributable cause of swelling.

**Figure 2:**
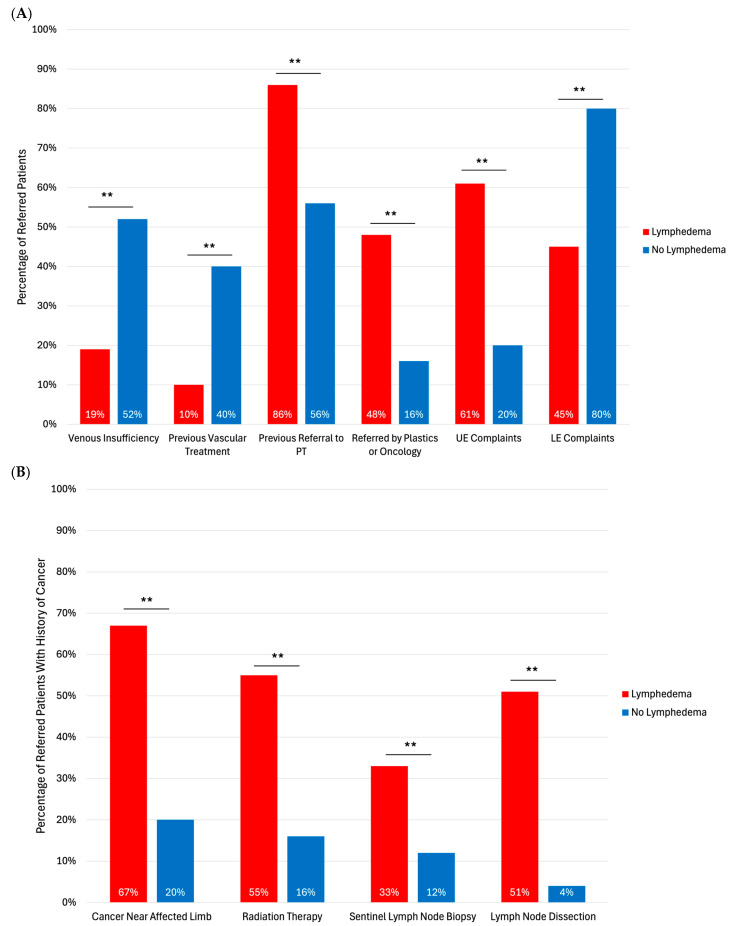
(**A**) Pre-referral correlates of lymphedema diagnosis confirmation. (**B**) Correlates of lymphedema diagnosis in patients with cancer. PT: Physical Therapy; UE: Upper Extremity; LE: Lower Extremity. Asterisks indicates statistical significance: ** *p* < 0.01.

Predictors of a confirmed diagnosis of lymphedema included previous physical therapy (86% vs. 56%, *p* < 0.01), referral by plastic surgery, surgical oncology, or medical oncology (48% vs. 16%, *p* < 0.01), and upper extremity symptoms (61% vs. 20%, *p* < 0.01). Patients without a confirmed diagnosis of lymphedema were more likely to have a history of venous insufficiency (52% vs. 19%, *p* < 0.01), prior vascular treatment (40% vs. 10%, *p* < 0.01), and lower extremity symptoms (80% vs. 45%, *p* < 0.01) (Figure 2A). Among patients with a history of cancer, those with confirmed lymphedema were more likely to have cancer near the affected limb (67% vs. 20%, *p* < 0.01), previous radiation therapy (55% vs. 16%, *p* < 0.01), previous sentinel lymph node biopsy (33% vs. 12%, *p* < 0.01), and previous lymph node dissection (51% vs. 4%, *p* < 0.01) (Figure 2B).

**Figure 3:**
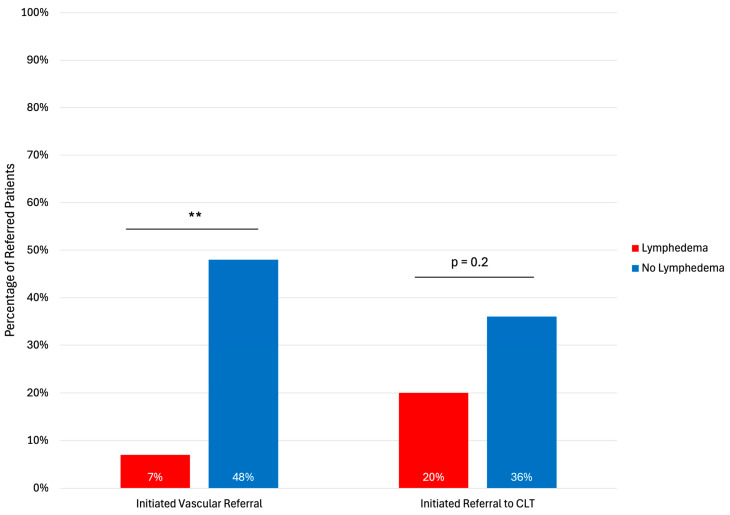
Secondary referrals for patients with and without lymphedema. CLT: Certified Lymphedema Therapist. Asterisks indicates statistical significance: ** *p* < 0.01.

We examined trends among secondary referrals (new referrals generated after consultation with a lymphatic surgeon) among patients with and without lymphedema. Patients without lymphedema were more likely to receive a secondary referral to vascular surgery compared to those with lymphedema (48% vs. 7%, *p* < 0.01) (Figure 3).

### 3.4. Surgical Candidates vs. Non-Surgical Candidates

**Figure 4:**
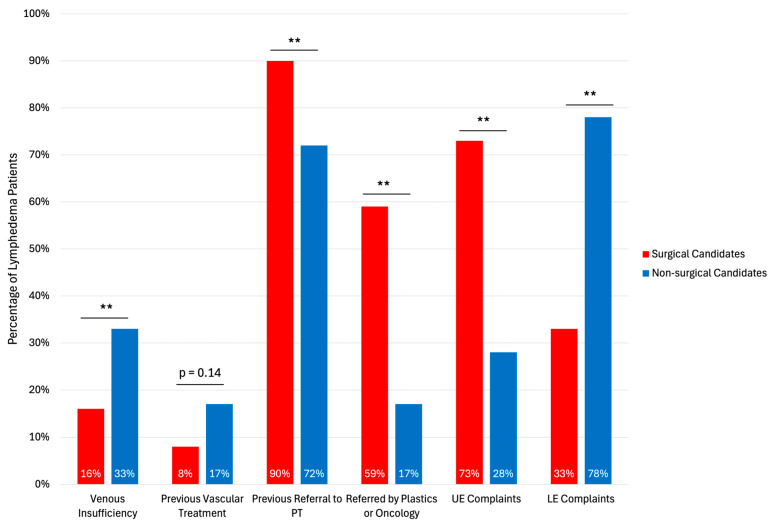
Pre-referral correlates of lymphatic reconstruction eligibility among patients with lymphedema. PT: Physical Therapy; UE: Upper Extremity; LE: Lower Extremity. Asterisks indicates statistical significance: ** *p* < 0.01.

Of the 69 patients diagnosed with lymphedema, 51 were candidates for microsurgical reconstruction, while 18 were not (Figure 1). Age was comparable between surgical and non-surgical candidates (58 ± 14 years vs. 63 ± 12 years, *p* = 0.19). Prior physical therapy (90% vs. 72%, *p* < 0.01), referral by providers familiar with lymphedema (plastic surgery, surgical oncology, medical oncology) (59% vs. 17%, *p* < 0.01), history of radiation therapy to the affected limb (65% vs. 28%, *p* < 0.01), previous lymph node dissection (61% vs. 22%, *p* < 0.01), upper extremity complaints (73% vs. 28%, *p* < 0.01), and lower BMI (27 kg/m^2^ vs. 34 kg/m^2^, *p* < 0.01) were correlated with surgical eligibility (Figure 4).

History of venous insufficiency (33% vs. 16%, *p* < 0.01), previous vascular treatment (17% vs. 8%, *p* = 0.14), and lower extremity complaints (78% vs. 33%, *p* < 0.01) were predictors of non-surgical eligibility.

**Figure 5:**
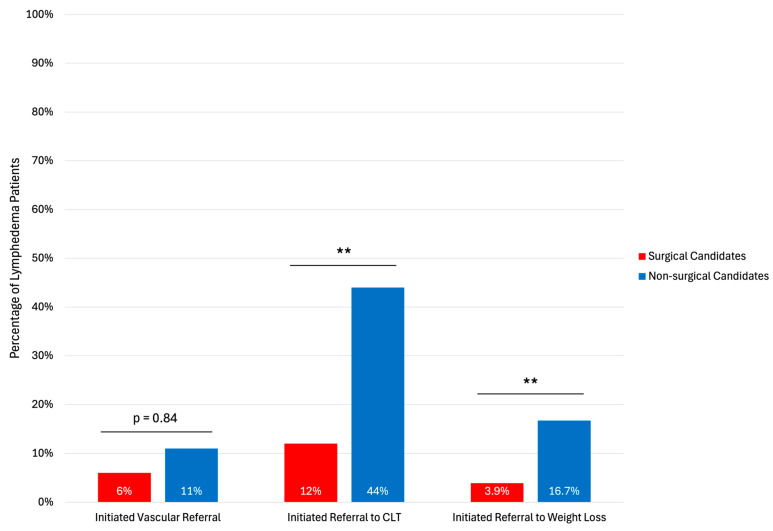
Secondary referrals for patients with confirmed lymphedema diagnosis. CLT: Certified Lymphedema Therapist. Asterisks indicates statistical significance: ** *p* < 0.01.

We examined secondary referrals for patients with a confirmed lymphedema diagnosis (Figure 5).

Surgical candidates were less likely to receive secondary referrals to CLT (12% vs. 44%, *p* < 0.01) or bariatric surgery (3.9% vs. 16.7%, *p* < 0.01). 6% of surgical candidates and 11% of non-surgical candidates received secondary referrals to vascular surgery (*p* = 0.84).

## 4. Discussion

### 4.1. Themes in the Correlates of Lymphedema Diagnosis and Surgical Eligibility

Our findings suggest that the accurate diagnosis of lymphedema after referral may be predicted by three categories of factors: (1) Direct anatomic injury to lymphatic structure, as seen in patients with a history of radiation, nodal dissection, and those who were referred by an oncologist. (2) Initial evaluation by providers familiar with lymphedema as seen in patients who were referred by plastic surgeons and oncologists, and those who had previously been seen by a CLT. (3) The absence of comorbidities that could also lead to swelling, as seen in patients with upper extremity symptoms, which are seldom related to venous insufficiency, and those without ongoing vascular treatment at the time of referral. Further supporting the notion that upper extremity symptoms highly suggestive of lymphedema are the results of one study, which reported a false positive rate of 51.5% vs. 11.9% among patients with swelling in their lower extremity vs. upper extremity [29].

Predictors of surgical candidacy followed similar patterns. Patients who had underlying lymphatic injury leading to anatomic obstruction from previous radiation therapy or lymph node dissection were more likely to be surgical candidates. Patients who had exhausted conservative treatments, such as those who had previously received physical therapy or had seen a CLT, were more likely to be surgical candidates. Patients with upper extremity symptoms and those referred by providers familiar with lymphedema, compared to those unfamiliar with lymphedema, also tended to be surgical candidates. This discrepancy clearly illustrates the potential for provider education to improve diagnosis and treatment.

A small proportion of surgical candidates were given secondary referrals to vascular surgery. There is significant overlap between venous and lymphatic pathology, as LVA relies on functioning, low-pressure veins to relieve lymphatic pressure and prevent backflow [30]. These patients represent the subset of microsurgical candidates who have incidental venous hypertension that must be corrected before being eligible for lymphatic microsurgery [30]. Traditional vascular techniques, such as venous ablation, can address superficial venous insufficiency but not deep venous insufficiency.

Patients with lymphedema who were non-surgical candidates had higher BMI and were more likely to receive secondary referrals to bariatric surgeons and medical weight loss specialists for OIL. Obesity not only increases the risk of lymphedema after lymphatic injury (surgery, radiation, trauma), but can also be an independent risk factor for developing lymphedema in the absence of surgery or prior interventions [22]. OIL is a distinct and recently recognized subtype of secondary lymphedema, presenting symmetrically, with no gross abnormalities visualized on lymphoscintigraphy. Because the primary lymphatic channels are normal and are physically obstructed by excess fatty tissue, lymphatic surgery is unlikely to be successful in patients with OIL [22]. Instead, the critical treatment is weight loss, whether it be surgically or medically achieved.

### 4.2. Tools for Disambiguation of Limb Swelling

**Figure 6:**
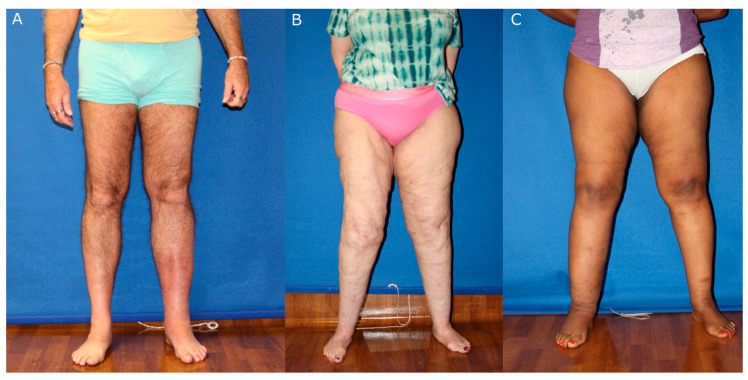
Clinical characteristics of diseases that confound the diagnosis of lymphedema. (**A**) Erythematous, tender, and indurated plaques characteristic of lipodermatosclerosis in the setting of venous stasis of the left lower extremity. (**B**) Symmetrical accumulation of fat sparing the feet, characteristic of lipedema. (**C**) Lymphedema of the right lower extremity affecting the foot with positive Stemmer sign.

Clinical examination is crucial during the initial triage of patients with swollen limbs (Figure 6). Venous stasis (Figure 6A) presents with hyperpigmentation and lipodermatosclerosis, with tender, indurated plaques in the early phase and woody, fibrotic induration in the late phase [31]. This clinical presentation overlaps with that of lymphedema, and the two pathologies may co-occur, but the presence of erythema and hyperpigmentation in band-like areas should raise suspicion for venous stasis [31]. Lipedema (Figure 6B) is also frequently mistaken for lymphedema; however, lipedema causes symmetrical accumulation of fat that spares the feet and may present with cuffing at the ankles [32]. The fat may be concentrated in the lower leg, the medial thighs, or centrally in the buttocks and hips, and typically has a “lumpy” appearance [32]. By contrast, lymphedema (Figure 6C) commonly affects the foot, and a positive Stemmer sign (inability to pinch the skin of the great toe) may be present.

**Figure 7:**
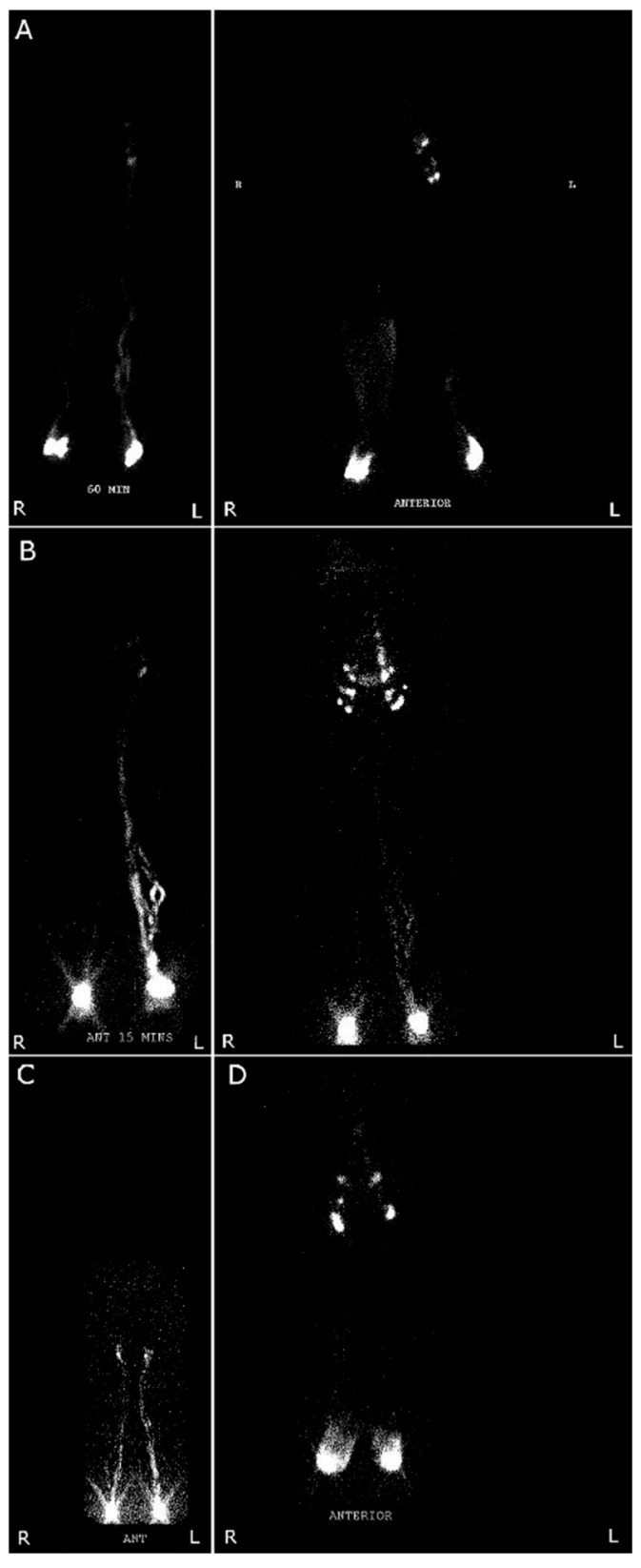
Anterior, lower body lymphoscintigraphy appearance of diseases that may be mistaken for lymphedema. (**A**) Lymphedema: delayed images (at 60 min and 5 h) with dermal backflow within the first hour and reduced visualization of lymphatics in the right leg. (**B**) Venous insufficiency with compensatory lymphatic activity: Images at 15 min and 4.5 h with immediate lymphatic drainage in both legs and more intense signal on the left in the setting of left-sided venous disease, suggesting. (**C**) Lipedema: Immediate image with slow transit time, without dermal backflow or structural abnormalities. (**D**) Obesity-induced lymphedema: Delayed image (4.5 h) with dermal backflow locally (greater on the right) and no evidence of structural or anatomic lymphatic abnormality. Abbreviations: L, Left; R, Right.

Imaging should be performed routinely to disambiguate the etiology of limb swelling. Venous duplex for valvular insufficiency is noninvasive and widely available, and should be performed with a tilt table or in a standing position [33]. Magnetic resonance lymphography and ICG lymphography are sensitive and specific, but lymphoscintigraphy is more widely available and may be performed near a patient’s home. Lymphoscintigraphic findings of lymphedema (Figure 7A) include the presence of dermal backflow and the failure of lymph nodes or lymphatic channels to opacify, especially in comparison to the normal limb [33]. In patients with venous insufficiency (Figure 7B), lymphoscintigraphy may reveal enhanced lymphatic flow in the affected limb as a compensatory mechanism [33]. Lymphoscintigraphy in lipedema (Figure 7C) demonstrates functional abnormalities with decreased transit speed, but reveals no structural abnormalities [32]. In OIL (Figure 7D), there is local dermal backflow, but no structural or anatomic abnormalities at the lymph nodes and lymphatic channels [22]. If the cause of the swelling is still unclear (for example, in localized post-traumatic lymphedema), ICG lymphography should be used to clarify the diagnosis. In real-world practice, overlap and potential misclassification among lymphedema, OIL, and lipedema may persist despite the application of diagnostic criteria. This is in part because obesity and lipedema can independently physically obstruct and impair lymphatic function, leading to secondary lymphedema [22]. Because these conditions often coexist, distinguishing among them can be challenging.

### 4.3. Patient Flow Models: Balancing Surgical Practice Resources with Patient Access to Care

Overall, our data illustrate that the majority of patients referred to a lymphatic surgery practice for limb swelling were not candidates for microsurgical reconstruction. Yet, over three quarters of these patients benefited from a referral to a specialist provider with the potential to treat and/or accurately diagnose their underlying problem. This is only possible when patients have access to lymphatic specialists without gatekeeping.

Older approaches to limb swelling evaluation relied primarily on clinical examination, with imaging such as lymphoscintigraphy or venous duplex ultrasound ordered selectively to confirm a suspected lymphedema diagnosis [34]. In contemporary multidisciplinary clinics, imaging is often ordered at the initial workup for new referrals and reviewed alongside clinical findings to confirm diagnosis and guide management and surgical candidacy [33,35]. Centers with referral criteria push forward the requirement for imaging before initial evaluation, effectively creating a barrier to evaluation by a specialist. Our approach is consistent with that of centers that order imaging at initial consultation, with the added advantage of a telemedicine-based initial evaluation to improve convenience and access for patients.

**Figure 8:**
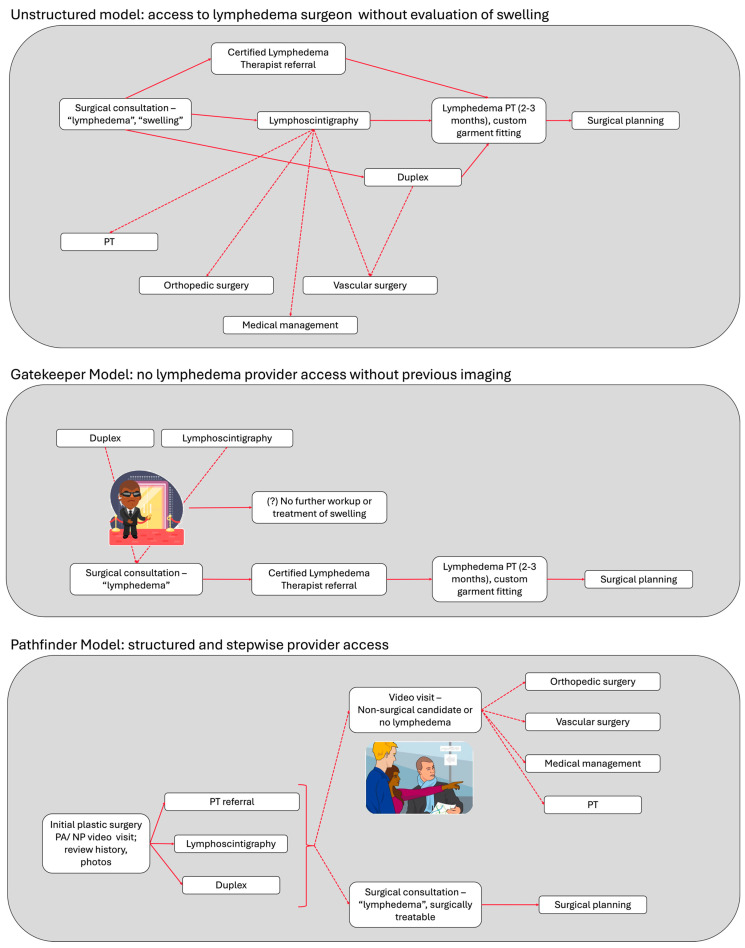
Flow diagrams of models for workup and treatment of swollen limb referrals to lymphedema centers: Unstructured; Gatekeeper; and Pathfinder. PT: physical therapy.

Figure 8 illustrates possible workflows for triaging swollen-limb referrals. In the *unstructured model*, the initial evaluation may result in surgical planning—if diagnostic workup is complete and the patient is a candidate—or in secondary referrals, which may or may not result in the ability to plan for physiologic reconstruction later. While this ensures patient have access to accurate diagnosis and treatment, it potentially misuses time, resources, and reduces the surgeon’s availability for other patients who are awaiting consultation. In the *gatekeeper model*, lymphoscintigraphy or equivalent imaging is required before referral, conserving surgeon time but risking under-referral and potentially leading to therapeutic nihilism among referring providers [25]. In the *pathfinder model*, an advanced practice provider within the lymphatic surgery program conducts an initial telemedicine screen, initiates duplex ultrasound and lymphoscintigraphy, and forwards the results for review by a microsurgeon. Patients with non-surgical conditions are then redirected to appropriate specialties, while surgical candidates proceed directly to an in-person consultation and ICG mapping.

In our study, the use of telemedicine for initial consultation was not associated with differences in diagnostic accuracy or in the determination of surgical candidacy compared to in-person evaluation. Although telemedicine became widely used during the COVID-19 pandemic and has been well studied in specialties such as dermatology, its role in lymphedema care remains relatively unexplored. Within the pathfinder model, telemedicine enhances patient access by facilitating timely referral, standardizing early clinical evaluation, and enabling appropriate ordering of diagnostic imaging. Telehealth-based and physiotherapist-based assessments of breast cancer-related lymphedema agree highly [36], with inability to assess skin texture and tone as the only limitation noted [37]. These advantages are particularly relevant for patients in under-resourced settings who may face barriers to timely or frequent evaluation.

The crux of the pathfinder model is the integration of a provider other than the lymphatic microsurgeon as part of the reconstructive microsurgery practice. This role is well suited for a nurse practitioner or physician assistant who works closely with the surgeon. Although currently only available at a few centers in the United States, lymphatic medicine physicians will also play an increasing role in the management of patients with limb swelling. Recruitment of specialized lymphologists to reconstructive practices can contribute to the deployment of emerging medical therapies for lymphatic disorders in addition to refining the application of existing therapies.

The implications of improving diagnostic and therapeutic workflows extend beyond logistics. Lymphedema is emblematic of a broader transformation in plastic surgery toward physiologic restoration and multidisciplinary integration. The transition from gatekeeper to pathfinder reflects the maturation of lymphatic surgery into a comprehensive discipline. As lymphatic reconstruction becomes more widespread, plastic surgeons will increasingly define standards for diagnosis, staging, and longitudinal care. The field’s future lies in maintaining accessibility while preserving rigor—ensuring that innovation serves patients not only through surgical precision but through systems that connect them efficiently to the right care. By embracing both the educational and reconstructive dimensions of care, plastic surgeons can shape the trajectory of lymphatic medicine within the evolving landscape of reconstructive microsurgery.

### 4.4. Limitations

The limitations of this study include its retrospective design, which prevents us from randomizing patients or establishing causation. Given that the data was collected from patients seen by a single surgeon at a single institution, we are unable to generalize these findings to the broader population of patients with lymphedema. We used lymphoscintigraphy as our routine diagnostic test for lymphedema. Although lymphoscintigraphy is still widely considered the gold standard for diagnosing lymphedema, other modalities may be more sensitive. The subjectivity inherent to determining surgical candidacy is another major limitation. We minimized potential bias by maximizing reliance on objective information- utilizing ICG lymphography, lymphoscintigraphy, and standardized criteria to determine OIL. Finally, our follow-up did not include lymphatic surgery or predictors of postoperative success such as limb volume, circumference, bioimpedance, or patient-reported outcome measures. Longer term follow-up is required to quantify the improvement in lymphedema following different surgical interventions.

## 5. Conclusions

Lymphedema is a commonly misunderstood and misdiagnosed disease. Despite advances in lymphatic reconstruction, patient care is hindered by inconsistent diagnostic practices and limited provider familiarity with the disease. Involving microsurgical reconstructive expertise in the care of patients with limb swelling results in improved diagnostic accuracy and referrals to specialists who can potentially offer new therapies, even for patients without lymphedema or who are not microsurgical reconstruction candidates. In a pathfinder model of patient care, advanced practice providers perform the initial consultation and workup, help triage patients who may be surgical candidates for surgical evaluation, and refer those who are not to other specialists. The pathfinder model avoids the use of referral criteria for gatekeeping and expands patient access to accurate diagnosis, without overwhelming surgical practices with a large volume of non-surgical patients.

## Figures and Tables

**Table 1:** Clinical and imaging characteristics of lymphedema-related and non-lymphedema-related causes of limb swelling.

**Lymphedema-Related Limb Swelling**						
**Etiology**	**Clinical Features**		**Location**	**Imaging Findings**	**Ancillary Tests**	
**Primary Lymphedema**						
Hereditary (30%) [26] Congenital/Idiopathic	Swelling **without inciting factor** (vascular obstruction/insufficiency, trauma, surgery, radiation) - Hereditary (30%) or sporadic		- Primarily lower extremity; unilateral or bilateral	- Positive lymphoscintigraphy *: dermal backflow, asymmetrical tracer uptake at nodes, or delayed transit time (>3 h)	- Genetic testing if syndromic features (verrucae, limb length asymmetry, atypical facial features, cognitive impairment, vascular malformations [7]	
**Secondary Lymphedema**						
Cancer-related	- Swelling related to cancer treatment (**radiation, lymph node dissection**)		- Variable depending on treatment site and extent of lymphatic dysfunction	- Positive lymphoscintigraphy *	None	
Phlebolymphedema	Swelling from **longstanding venous hypertension or obstruction** - Varicosities, hyperpigmentation, lipodermatosclerosis, ulcers		- Lower >> upper extremities; unilateral or bilateral	- Positive lymphoscintigraphy * - **Venous duplex with obstruction or reflux, or venography with central vein stenosis or compression**	None	
Post-traumatic/ Iatrogenic	- Prolonged swelling **after infection, compartment syndrome, trauma, or surgical procedures**		- Variable depending on the location of the inciting event	- Positive lymphoscintigraphy *	- ICG imaging, if normal lymphoscintigraphy and localized symptoms [27]	
Obesity-Induced Lymphedema (OIL)	- Body mass index > **40 kg/m^2^**		Lower extremities; bilateral - Or, massive localized lymphedema [22]	- Lymphoscintigraphy: **patent lymphatic channels, normal inguinal nodes, and distal or localized dependent dermal backflow**	None	
Lipolymphedema	- Features of lipedema (below) + **mixed pitting and nonpitting edema**		- Symmetrical, lower > upper extremities	- Lymphoscintigraphy: **dermal backflow with normal inguinal lymph nodes**	None	
**Non-Lymphedema-Related Limb Swelling**						
**Clinical Features**		**Location**		**Imaging Findings**	**Ancillary Tests**	
**Lipedema**						
- Tender lipodystrophy - Ankle cuffing, **sparing feet** - Swelling not improved by elevation - Weight-loss resistant		- Bilateral and symmetrical, disproportionately affecting the lower extremities		- Lymphoscintigraphy: **normal or structurally normal with delayed lymphatic transit time**	- MRI: fat predominant without edema	
**Chronic Venous Insufficiency**						
Pruritus, stinging pain, cramps - Varicose veins, erythema, ulcers, darkening of skin over shin, lipodermatosclerosis		- Bilateral or unilateral lower extremities		- Lymphoscintigraphy: symmetrical lymphatic drainage with no dermal backflow - Duplex: **multisegment superficial reflux > 500 ms, or multisegment deep reflux > 1000 ms**	None	

Note: Bolded text highlights key features. Asterisk indicates imaging findings for positive lymphoscintigraphy apply to subsequent rows unless otherwise specified.

**Table 2:** Patient demographics and location of reported symptoms.

**Patient Characteristics**		***n* (%)**
Sex	Male	26 (27.7%)
	Female	68 (72.3%)
Age (years)		61 ± 14
Body Mass Index (kg/m^2^)		29.8 ± 8
**Past Medical History**		***n* (%)**
Deep vein thrombosis		12 (12.7%)
Venous insufficiency		26 (27.7%)
Smoking status	Former	25 (26.6%)
	Current	6 (6.4%)
**Chief Complaint Location**		***n* (%)**
Upper extremity	Left	28 (29.8%)
	Right	21 (22.3%)
	Bilateral	2 (2.1%)
	Overall	47 (50%)
Lower extremity	Left	41 (43.6%)
	Right	40 (42.6%)
	Bilateral	30 (31.9%)
	Overall	51 (54.3%)
Upper and lower extremity		4 (4.3%)

**Table 3:** Clinical characteristics of patients referred for lymphedema.

**Cancer Characteristics**	***n* (%)**
Cancer near the affected limb	51 (54.3%)
Prior radiation therapy	42 (44.7%)
Prior sentinel lymph node biopsy	26 (27.7%)
Prior lymph node dissection	36 (38.3%)
Prior chemotherapy	31 (32.9%)
**Physical Therapy (PT) and Certified Lymphedema Therapist (CLT)**	***n*** **(%)**
Previous non-lymphedema related PT attended	70 (74.5%)
Previously seen CLT	73 (77.7%)
Currently seeing CLT	51 (54.3%)

**Table 4:** Distribution of primary referral sources for lymphedema by specialty.

Referral Source	*n* (%)
Plastic and Reconstructive Surgery	20 (21.2%)
Orthopedic Surgery	13 (13.8%)
Self-Referred	10 (10.6%)
Medical Oncology	8 (8.5%)
Physical Therapy	8 (8.5%)
Primary Care	7 (7.4%)
Breast Surgery	7 (7.4%)
Vascular Surgery	6 (6.4%)
Physical Medicine and Rehabilitation	4 (4.3%)
Radiology	4 (4.3%)
Obstetrics & Gynecology	2 (2.1%)
Nephrology	1 (1.1%)
Podiatry	1 (1.1%)
Otorhinolaryngology	1 (1.1%)
Occupational Therapy	1 (1.1%)
Surgical Oncology	1 (1.1%)

## Data Availability

Data are available at reasonable request.

## Abbreviations

The following abbreviations are used in this manuscript:

ICG	Indocyanine green
OIL	Obesity-induced lymphedema
CLT	Certified lymphedema therapist
PT	Physical therapy
